# Beginning the New Volume

**Published:** 1917-08

**Authors:** 


**A Monthly Review of Medicine and Surgery**

EDITOR AND PUBLISHER, DR. A. L. BENEDICT, 228 Summer St., corner of Elmwood Ave., Buffalo, (Address for all communications. Please make personal and telephone calls before 1 P. M.)

**Third, in age, on the western continent. Independent, but supports professional organizations. News limited mainly to Western N. Y. and Penn.**

**Subscription $2.00 a year; $3.00 for two years in advance. Changes and errors in address or failure or duplication of delivery, should be reported immediately.**

Yearly Volume 73 AUGUST, 1917 Number 1

### Beginning the New Volume.

In spite of general business depression shortly after the assumption of the JOURNAL six years ago and some disturbances due to the world war, we are happy to report very satisfactory progress. The number of subscribers has rather more than doubled and the only source of loss worth considering is death. Local gains are necessarily small since the medical profession is almost at a standstill so far as numbers are concerned—a very salutary fact in view of the past excessive graduation of physicians—and since the local support has always been strong. Especially gratifying to all contributors, is the continued though moderate increase in subscriptions from a distance which excludes the idea of performing a duty and a purely local interest.

A journal of this nature cannot and, perhaps, should not be highly successful financially. The conduct of a medical journal by men not actively engaged in practice, involves a point of view which is extra-professional and, thus, a lack of sympathetic judgment in accordance with the actual standards of the profession. The JOURNAL has, however, for some time, carried a reserve covering two issues in advance and could cover 6 issues in advance if a comparatively small number of subscribers in arrears for several years, would view matters from the standpoint of the welfare of the JOURNAL instead of their own immediate convenience.

It is an open secret which should be no secret at all, that the major support of any periodical, excepting a few which are virtually endowed by private individuals or large organizations, a few popular magazines of large circulation, and a few special organs of high price, is derived from advertising. This statement applies not only to medical journals but to newspapers and periodicals generally. It should be understood with equal clearness that the principal difficulty in the conduct of any periodical is the competition of the circular letter. Firms which have checked up the actual value of the circular, estimate that the returns are from ½ to 3%. This is a small ratio but many firms, or at least their advertising managers, prefer actual figures to general impressions. Hence, we ask, not for this journal alone, nor for medical journals as a class, but for all periodicals, that readers should indicate the source of their information. If an advertisement is keyed, ask for catalogue 25659, or address to Dept. K, or insert the street address, even if you know that catalogue 25659 is precisely the same as No. 24371, or that Dept. K simply means that the reply is from the paper in which you read the advertisement, or that the street address is unnecessary to reach the addresses or incorrect—or, perhaps better yet, ignore the key and state that you saw the ad. in such and such a periodical. Most busy men have a convenient file for circulars, woven from willow twigs. Use it and let advertisers know that it is in common use.

A journal of this kind obviously cannot maintain a press bureau nor a reportorial staff. We, therefore, ask the cooperation of the profession in communicating news items, especially of deaths in the profession.

